# Neuropsychological profiles of adult bipolar disorder patients with and without comorbid attention-deficit hyperactivity disorder

**DOI:** 10.1186/s40345-019-0149-9

**Published:** 2019-06-28

**Authors:** Sara Salarvan, Timea Sparding, Caitlin Clements, Eleonore Rydén, Mikael Landén

**Affiliations:** 1 Department of Psychiatry and Neurochemistry, Institute of Neuroscience and Physiology, The Sahlgrenska Academy, University of Gothenburg, Sahlgrenska University Hospital, Blå Stråket 15, 413 45 Gothenburg, Sweden; 2 Department of Psychology, University of Pennsylvania, 3720 Walnut Street, Philadelphia, PA 19104 USA; 3 Department of Medical Epidemiology and Biostatistics, Karolinska Institutet, Stockholm, Sweden; 4 Center for Autism Research, The Children’S Hospital of Philadelphia, 2716 South St, Philadelphia, PA 19104 USA; 5 Department of Clinical Neuroscience, Karolinska Institutet, Stockholm, Sweden

**Keywords:** Attention-deficit/hyperactivity disorder, Bipolar disorder, Comorbidity, Cognitive function, Neuropsychology

## Abstract

**Background:**

Comorbid attention-deficit/hyperactivity disorder (ADHD) is common in bipolar disorder and associated with worse outcomes. Cognitive testing might be a tool to identify this group. Here we compare the neuropsychological profiles of bipolar disorder patients with (BD + cADHD) and without (BD − cADHD) childhood attention-deficit hyperactivity disorder.

**Methods:**

Adult patients with BD  −  cADHD (n = 66), BD + cADHD (n = 32), and healthy controls (n = 112) were tested using a comprehensive battery of neuropsychological tests. Patients underwent rigorous diagnostic assessments for bipolar disorder and ADHD, as well as a parental interview to establish childhood ADHD.

**Results:**

The neuropsychological profiles of the groups were similar, except that the BD + cADHD group performed significantly worse on working memory. Working memory did not differ between those in the BD + cADHD group who only had a history of childhood ADHD and those that still met criteria for ADHD in adulthood.

**Conclusions:**

Cognitive testing had limited power to differentiate between bipolar disorder adults with and without childhood ADHD. The BD + cADHD subgroup cannot explain the significant cognitive heterogeneity seen in bipolar disorder patients.

**Electronic supplementary material:**

The online version of this article (10.1186/s40345-019-0149-9) contains supplementary material, which is available to authorized users.

## Introduction

The prevalence of comorbid attention-deficit/hyperactivity disorder (ADHD) in adults with bipolar disorder (BD) ranges between 9.5 and 28% (Nierenberg et al. 2005; Rydén et al. 2009; Torres et al. 2015). Although the longitudinal course of illness differs between BD and ADHD, where BD is an episodic disorder and ADHD is a persistent condition, differentiating between ADHD and BD is sometimes a challenge (Brus et al. 2014). BD and ADHD feature partially overlapping symptoms that cross-sectionally might be interpreted as hypomanic symptoms, such as talkativeness, restlessness, impulsivity, distractibility, and affective lability.

The overlap of symptoms not only begets diagnostic challenges, it also raises the question if so-called ‘comorbid’ BD and ADHD condition might be an artefact produced by the diagnostic system (Wingo and Ghaemi 2007). The comorbidity concept is, however, supported by the fact that a majority of patients with comorbid BD and ADHD maintain both diagnoses after correction for overlapping symptoms (Milberger et al. 1995). Moreover, BD with comorbid ADHD presents a worse course of illness than BD without ADHD, including earlier onset of bipolar disorder (Nierenberg et al. 2005; Tamam et al. 2008; Rydén et al. 2009; Karaahmet et al. 2013; Perroud et al. 2014), as well as higher frequency of mood episodes (Nierenberg et al. 2005; Tamam et al. 2008; Rydén et al. 2009; Perroud et al. 2014; Torres et al. 2015), suicide attempts (Nierenberg et al. 2005; Torres et al. 2015; Harmanci et al. 2016), and interpersonal violence (Nierenberg et al. 2005; Rydén et al. 2009). This means that it is clinically important to identify BD patients with comorbid ADHD. It is noteworthy that the mere history of childhood ADHD, irrespective of adult ADHD status, is sufficient to generate a worse course of illness in BD (Rydén et al. 2009; Torres et al. 2015). This suggests that reviewing childhood history is important when assessing BD prognosis.

BD has been associated with general cognitive impairment across different cognitive domains (Mann-Wrobel et al. 2011; Sparding et al. 2015). Meta-analyses suggest that executive function, verbal memory, and attention/working memory are particularly affected (Robinson and Ferrier 2006; Torres et al. 2007; Arts et al. 2008; Bourne et al. 2013). However, more recent studies suggest that cognitive impairment may not be a general trait in BD, but limited to specific subgroups (Burdick et al. 2014; Bora et al. 2016; Sparding et al. 2017): one group of patients that perform within the normal range, and one or more subgroups that exhibit varying degrees of cognitive impairment. It remains unknown what accounts for this cognitive heterogeneity in BD, but a possible explanation is that the cognitively impaired group suffer from comorbid conditions, e.g., ADHD. Cognition in adults with ADHD shows general impairments across multiple cognitive domains, with possible emphasis on impaired attention, inhibition, and verbal memory (Hervey et al. 2004; Schoechlin and Engel 2005). Despite this, studies of cognitive function in BD have generally not accounted for potential comorbid ADHD.

Examining cognition in BD patients with and without comorbid ADHD might also serve to identify a BD subgroup with poor outcome in need for specific interventions. One study compared adults with BD, ADHD, BD + ADHD, and healthy controls across a broad spectrum of cognitive domains, but found no significant differences between BD and BD + ADHD (Torres et al. 2017). However, the assessment of comorbid ADHD did not include any informant report. The accuracy of retrospective self-reports of childhood symptoms has been questioned (Barkley et al. 2002; Mannuzza et al. 2002), and it is considered best practice to include multiple sources of information, e.g., a parental or next of kin interview (Davidson 2008). Furthermore, replication is needed given the limited sample size in the previous study (Torres et al. 2017).

The aim of the present study was to compare the neuropsychological profiles of adult BD patients with childhood ADHD (BD + cADHD) and without childhood ADHD (BD − cADHD) in a cohort with meticulous assessment of childhood as well as adult ADHD.

## Materials and methods

The participants in this study were recruited from a bipolar outpatient clinic at the Northern Stockholm psychiatric clinic in Sweden between October 2005 and April 2008, as a part of the St. Göran Bipolar project. The project and the clinical assessment process has been described in detail elsewhere (Rydén et al. 2009). In brief, the project is a prospective, longitudinal study providing assessment, treatment, and follow-up of patients with BD. For the present study, we included patients who were at least 18 years of age, met the DSM-IV criteria for BD type I or II, and consented orally and in writing to participate in the project. Exclusion criteria were a history of intellectual disability (IQ < 70), organic brain disorder, neurological disorder, and severe medical conditions. The study was approved by the ethical review board in Stockholm, Sweden.

### Assessment of bipolar disorder

The baseline clinical assessment was made by a psychiatrist or a resident in psychiatry using the affective disorder evaluation (ADE), a diagnostic instrument originally developed and used in the systematic treatment enhancement program of bipolar disorder (STEP-BD) (Sachs et al. 2003) that had been slightly modified to suit Swedish conditions. The ADE includes the affective module of the Structural Clinical Interview for DSM-IV (SCID). The Mini International Neuropsychiatry Interview (M.I.N.I) (Sheehan et al. 1998) was used to screen for other psychiatric diagnoses. Other information gathered with the ADE include a social anamnesis, number of lifetime affective episodes and their characteristics, alcohol and drug abuse, somatic illnesses, histories (childhood, family, and treatment), and suicide attempts.

Patients completed two self-report questionnaires to screen for alcohol and substance abuse: the Alcohol Use Disorders Identification Test (AUDIT) (Saunders et al. 1993) and the Drug Use Disorders Identification Test (DUDIT) (Berman et al. 2005). Taking information from all available sources into account, the final diagnostic decision was made at a diagnostic case conference by a consensus panel of experienced board-certified psychiatrists specialized in bipolar disorder.

### Assessment of ADHD

The assessment of childhood and current ADHD symptoms was completed after the bipolar diagnosis had been established and at a time when the patient was considered stable with respect to mood symptoms (but not always completely asymptomatic) by the treating physician. The ADHD clinical assessments required approximately 1.5 h and were conducted by a second independent board-certified psychiatrist (E.R.). To assess current ADHD symptoms, the Adult ADHD Self-Report Scale (ASRS) (Kessler et al. 2005) and the BROWN ADD assessment scale (Brown 2008) were used. To retrospectively assess childhood symptoms of ADHD, the Wender Utah Rating Scale (WURS-25) (Ward et al. 1993) was used. To obtain objective information about childhood symptoms, the Autism-Tics, ADHD, and other comorbidities (A-TAC) interview was completed with a parent or other next of kin in a telephone interview when possible. The A-TAC is a broad screening instrument to assess child psychiatric disorders with a particular strength in assessing ADHD and autism spectrum disorders symptoms (Mårland et al. 2017). The A-TAC interview took approximately 30 min to complete.

### *Pure bipolar group, and bipolar disorder* + *childhood ADHD group*

The present study compared the neuropsychological profiles of two groups:

1. *The bipolar disorder and childhood ADHD group (BD* + *cADHD)* (n = 32) fulfilling
  - criteria for bipolar disorder type I or II, and
    i. criteria for childhood ADHD alone (defined as an A-TAC ADHD score ≥ 8, and ⁄or a WURS-25 score ≥ 46) (n = 14), or
    ii. criteria for childhood *and* current ADHD (defined as meeting childhood ADHD criteria above, and currently meeting DSM-IV criteria for any of the three subtypes of ADHD) (n = 18)
2. *The bipolar disorder without childhood ADHD group (BD − cADHD)* (n = 66) fulfilling criteria for bipolar disorder type I or II without a history of childhood ADHD.

### Healthy control group

In supplementary analyses, we also included a healthy control group randomly selected by Statistics Sweden. Details of the recruitment, in- and exclusion criteria can be found elsewhere (Rolstad et al. 2015; Abe et al. 2018). Briefly, eligible controls were scheduled for a personal examination by a psychiatrist using M.I.N.I. and selected parts of the ADE. Exclusion criteria for controls were any current psychiatric disorder, a family history of schizophrenia or bipolar disorder in first-degree relatives, drug or alcohol abuse, and neurological conditions.

### Neuropsychological test procedure

The baseline assessments of the St. Göran Bipolar project consisted of a neuropsychological test battery administered by a trained psychologist according to standard instructions. The testing usually required 2 sessions to complete. Patients were tested in a stable mood (but not always completely asymptomatic) as judged by the treating physician. Present mood status was assessed by administrating the Montgomery–Åsberg Depression Rating Scale (MADRS) (Åsberg and Schalling 1979) and the Young Mania Rating Scale (YMRS) (Young et al. 1978). Patients with > 14 on either scale were not considered to be in clinically stable mood and excluded from this study.

The neuropsychological assessment included a broad set of cognitive tests routinely employed in clinical settings (Lezak 2012): Wechsler Adult Intelligence Scale, third edition (WAIS-III) measuring intellectual ability; Rey Complex Figure test (RCFT) primarily measuring visuospatial memory; Claeson Dahl Verbal Learning and Retention test measuring verbal memory; Conners’ Continuous Performance Test 2 (CPT 2) measuring different aspects of attention; and five tests from the Delis-Kaplan Executive Function System (D-KEFS) measuring different aspects of executive functions (Trail Making Test, Design fluency test, Verbal Fluency Test, Color-Word Interference Test, Tower Test).

For the current study, we selected 10 subtests that cover a broad range of cognitive domains and identify the largest systematic variation in this cohort of patients with bipolar disorder (Sparding et al. 2015, 2017). These tests were: Verbal Comprehension, Perceptual Organization, Working Memory and Processing speed indexes from WAIS-III; Commission- and Omission errors from CPT 2 (measuring concentration/focused attention); Color Word 4 from D-KEFS (measuring cognitive flexibility); and Total achievement Score on Tower Test (measuring planning and decision making).

### Statistical analyses

All data were analysed with SPSS version 23. The groups were compared with t-tests for continuous variables, and chi-square tests for categorical variables. Equal variances between groups was tested with Levene’s test and corrected if p < 0.05. Effect sizes were calculated with Cohen’s *d* value (where 0.2 corresponds to small, 0.5 to moderate, and 0.8 to large effect size). We also conducted complementary analyses comprising a healthy control group (Additional file 1: Tables S1a and S2). We compared the three groups using one-way analysis of variance (ANOVA). Specific group differences were examined with post-hoc Bonferroni tests. Statistical significance was set at a two-sided p-value of < 0.05.

## Results

The clinical characteristics of the BD − cADHD and the BD + cADHD groups are shown in Table 1. The groups did not differ with respect to sex, age, type of bipolar disorder, or global IQ (as measured by WAIS-III). The prevalence of comorbid psychiatric diagnoses other than ADHD was numerically (but not statically significant, p = 0.09) higher in the BD + cADHD group compared with the BD − cADHD group. Specific comorbidities in each group are outlined in Additional file 1: Table S1b. The BD + cADHD group was significantly younger at age of first psychiatric symptom, as well as at age of first affective episode. The number of mixed episodes was significantly higher for the BD + cADHD group, whereas no group differences were found regarding number of manic, hypomanic, or depressive episodes. Lithium treatment was significantly more common in the BD − cADHD group, while other mood stabilizers were more common in the BD + cADHD group. No differences were found regarding level of education, employment status, or number of sick-leave days the previous year.

**Table 1:** Clinical characteristics of the bipolar disorder group without childhood ADHD (BD − cADHD) and the bipolar disorder group with childhood ADHD (BD + cADHD)

	BD − cADHD (*n* = 58–66^a^ )	BD + cADHD (*n* = 24–32^a^)	*p*-value
Male, N (%)	28 (42.4)	15 (46.9)	.68
Female, N (%)	38 (57.6)	17 (53.1)	
Age, mean (SD)	37.7 (13.6)	35.2 (11.5)	.36
Age at first psychiatric symptom, mean (SD)	20.4 (8.4)	14.8 (10)	.01
Age at first affective episode, mean (SD)	20.9 (8.1)	16.0 (7.1)	.01
Bipolar I, N (%)	44 (67.7)	15 (55.6)	.27
Bipolar II, N (%)	21 (32.3)	12 (44.4)	
Any comorbid psychiatric diagnosis, N (%)	36 (58.1)	20 (76.9)	.09
AUDIT, mean (SD)	5.5 (4.5)	6.6 (7)	.49
DUDIT, mean (SD)	0.9 (2.5)	2.6 (6.6)	.22
MADRS, mean (SD)	4.6 (4)	3.9 (3.3)	.40
YMRS, mean (SD)	1.3 (2.1)	2 (2.7)	.16
No of mood episodes, mean (SD)			
Mania	1.9 (2.7)	1.7 (2.9)	.81
Hypomania	4.3 (7.7)	7.3 (11.8)	.16
Mixed	0.4 (1.8)	6 (10.4)	.01
Depressive	8.9 (10.9)	16 (21.1)	.11
History of attempted suicide and/or self-harm, N (%)	22 (34.4)	13 (48.1)	.22
Pharmacological treatment, N (%)			
Lithium	46 (70.8)	11 (36.7)	.00
Other mood stabilizers	13 (20)	15 (50)	.00
Antidepressant	24 (36.9)	10 (33.3)	.73
Antipsychotics	13 (20)	5 (16.7)	.70
Central stimulants	0	0	
WAIS-III estimated IQ	107.9 (16)	106.4 (10.9)	.60
At least 2 years university education, N (%)	38 (58.5)	15 (55.6)	.80
Working, N (%)	47 (73.4)	18 (66.7)	.51
Sick-leave days previous 12 months, mean (SD)	116.7 (145.6)	91.3 (134.8)	.45

^a^Data were missing for some patients, therefore the N varies

### Neuropsychological profiles

The results of the neuropsychological test scores are shown in Table 2. The BD-cADHD group performed significantly better than the BD + cADHD group on WAIS Working Memory Index (t (89) = 2.15, p = 0.03) with a moderate effect size (d = 0.52). For all other measures, the group differences were much smaller (d = − 0.02  to 0.29) and no other group differences were statistically significant.

**Table 2:** Comparison of neuropsychological test performance of the bipolar disorder group without childhood ADHD (BD − cADHD) and the bipolar disorder group with childhood ADHD (BD + cADHD)

	BD − cADHD (*n* = 56–64^a^)	BD + cADHD (*n* = 23–31^a^)	*t*-value	*p *value	Cohen’s *d*
	Mean (SD)	Mean (SD)			
WAIS-III: Verbal Comprehension Index	109.8 (12.9)	112.5 (10.6)	− .98	.33	− .23
WAIS-III: Perceptual Organization Index	109.4 (16.5)	106.1 (14.4)	.91	.37	.21
WAIS-III: Working Memory Index	102.2 (14.9)	95.4 (11)	2.15	.03	.52
WAIS-III: Processing Speed Index	99.1 (15.8)	95.9 (10.9)	1.12	.27	.24
CPT2 omission errors^b^	53.5 (15.8)	53.9 (16.7)	− .10	.92	− .02
CPT2 comission errors^b^	54.2 (9.9)	54.4 (10.1)	− .05	.96	-.02
ColorWord 4 (Inh/Swi)	9.9 (3)	9.5 (3.1)	.66	.51	.13
Tower test total	11.4 (3.3)	10.4 (3.7)	1.17	.25	.29
RCFT: immediate recall	43.5 (14.2)	41.6 (16)	.70	.49	.15
Claeson Dahl verbal learning	46 (12.9)	48.8 (9.3)	− 1.15	.25	− .25

^a^Data were missing for some patients, therefore the N varies

^b^Higher score indicates better performance on all tests except CPT, where lower score indicates better performance

In a posthoc analysis, we split the BD + cADHD group into individuals fulfilling only childhood ADHD criteria (n = 11) and individuals fulfilling both childhood and current ADHD criteria (n = 17). We compared the WAIS Working Memory Index but found no difference [mean (SD) 94.6 (11.9), and 95.9 (10.8), respectively, t (26) = − 0.32, p = 0.75]. The N differs from the main analysis because some individuals did not have complete data on the WAIS working memory variable.

In supplementary analyses, we compared the patient groups with a healthy control group to show the severity of cognitive impairment (Additional file 1: Table S2). Results show that both patient groups performed worse than controls on several tests. There was a significant difference across all three groups with respect to Working memory, but the difference between the two bipolar subgroups was not significant in the post-hoc Bonferroni test. Note that comparisons of cognitive function between cases and controls in this cohort have been published previously (Pålsson et al. 2013; Sparding et al. 2015).

## Discussion

This study compared the neuropsychological profiles of adult BD patients with and without comorbid childhood ADHD. We payed particular attention to childhood history and performed interviews with parents or next of kin to establish childhood ADHD diagnoses. The main finding was that the neuropsychological profiles of the groups were similar. The only exception was that the BD + cADHD group performed significantly worse on WAIS working memory than the BD − cADHD group, but that finding would not withstand correction for multiple testing (N = 10).

There are few previous studies in this area. Our finding of small overall cognitive differences between BD-cADHD and BD + cADHD is largely in agreement with the results of Torres and colleagues (Torres et al. 2017) who found no significant differences between BD and BD + ADHD. Another study (Silva et al. 2014) used only the Wisconsin Card Sorting Test and found that BD + ADHD showed set-shifting difficulties compared with ADHD and healthy controls. One study comparing adolescents with BD, ADHD, BD + ADHD, and healthy controls (Rucklidge 2006) found that the BD group in general performed similar to controls, whereas BD with comorbid ADHD was associated with cognitive impairment. The greatest deficits in the BD + ADHD group were found in verbal memory and inhibitory control. Working memory was impaired only in the ADHD group.

It is noteworthy that the few studies comparing BD and ADHD with respect to cognition suggest that differences were directly or indirectly attributable to working memory impairment in ADHD (Torralva et al. 2011; Baez et al. 2014). With the caveat that our results were not corrected for multiple comparisons, the finding of worse working memory of moderate effect size in the BD + cADHD group is interesting because conceptual models of ADHD place deficits in working memory as either a core feature of the disorder (Rapport et al. 2001), or as an area of secondary but fundamental impairment (Barkley 1997). In fact, a meta-analysis examining working memory in adults with ADHD found deficits of moderate effect size and concluded that working memory impairment in ADHD persist into adulthood (Matt Alderson et al. 2013).

Drilling deeper into the BD + cADHD group, we found no difference in working memory between those who met criteria for ADHD in childhood as well as adulthood, and those who only did so in childhood. This indicates that adults with BD with a history of childhood ADHD are likely to show working memory impairments regardless of current ADHD status.

Our findings have three important clinical implications. First, previous studies have found that BD with comorbid ADHD is associated with worse course of illness, calling for instruments to readily identify this group clinically (Rydén et al. 2009; Torres et al. 2015). Unfortunately, this study suggests that cognitive testing is not likely to be a valuable tool to differentiate between BD with and without ADHD given that we found quite similar neuropsychological profiles. This might be surprising since cognitive testing is considered to be an essential source of information in ADHD assessment. But there is in fact no agreed upon cognitive profile specific for ADHD (Davidson 2008), nor is there consensus on a testing battery that would identify cognitive impairments in BD [although there have been suggestions (Yatham et al. 2010; Sparding et al. 2015)]. This absence of disorder-specific cognitive profiles might be due to inherent limitations with current neuropsychological batteries or that cognitive impairments are orthogonal to psychiatric syndrome diagnoses.

Second, our results show that childhood ADHD was associated with impaired working memory in BD regardless of whether the diagnosis persisted into adulthood or not. This aligns with studies indicating that the mere history of childhood ADHD is sufficient to generate a worse course of bipolar illness (Rydén et al. 2009; Torres et al. 2015). Other studies have found cognitive impairment in BD to be associated with worse course of illness (Robinson and Ferrier 2006; Bourne et al. 2013)—with the highest correlation for working memory—and poorer everyday functioning (Depp et al. 2012). Hence, future research could investigate in more detail if cognitive testing focusing on working memory together with a thorough assessment of childhood symptoms of ADHD might be of value in identifying a group of BD patients at risk for poor outcomes.

Third, cognitive impairments observed in BD patients as a group have been attributed to one or more subgroups with more pronounced impairment (Burdick et al. 2014; Bora et al. 2016; Sparding et al. 2017). So far, no proposed characteristics of these cognitive subgroups have been able to explain the cognitive variability, i.e., bipolar subtype (Pålsson et al. 2013; Bora 2018), prior psychotic manifestations (Savitz et al. 2009; Bora 2018), number of affective episodes (Mann-Wrobel et al. 2011), residual mood symptoms, or medication (Bourne et al. 2013). Our findings suggest that nor is comorbid ADHD likely to explain the heterogeneous cognitive performance in BD beyond working memory deficits.

Some limitations of this study should be considered. First, although the sample size was on par with previous studies in the area, a limited number of study subjects might have affected the possibility to detect group differences with ensuing risk of type II errors. Second, we did not correct for multiple comparisons; doing so would revoke the group difference in working memory. Moreover, in the supplementary analyses where a healthy control group was added, working memory was no longer statistically significantly between the bipolar disorder groups. Third, ADHD is a childhood onset diagnosis that in this study was made in retrospect. Even though we used validated instruments to assess childhood symptoms, conducted a separate diagnostic interview focusing on ADHD, and even included an interview with next-of-kin when possible, this study design is susceptible to recall bias. Fourth, although not statistically significant, there were more BD II patients in the BD + cADHD group. We did not find any differences between BD I and BD II in a previous study (Sparding et al. 2015,), but a recent meta-analyses found that BD I performed significantly worse than BD II with respect to global cognition, verbal memory, processing speed, and executive functioning speed and accuracy (Bora 2018). Fifth, the use of mood stabilizers differed such that lithium was more common in the BD − cADHD group whereas other mood stabilizers (e.g., valproate, lamotrigine) were more common in the BD + cADHD group. It cannot be excluded that medication might affect cognitive performance.

## Conclusions

Cognitive performance was similar in bipolar disorder patients with and without ADHD in childhood, except that the former group performed worse on working memory. This means that comorbid ADHD cannot explain the cognitive heterogeneity seen in bipolar disorder patients, and that cognitive testing is not likely to be useful for differentiating between BD with and without ADHD.

## Additional file

**Additional file 1.** Additional tables.

## Data Availability

The data that support the findings of this study are available on reasonable request from the corresponding author (ML). The data are not publicly available due to information that could compromise research participant privacy.
