# Supplementary material for: Neuropsychological profiles of adult bipolar disorder patients with and without comorbid attention-deficit hyperactivity disorder
Source: Int J Bipolar Disord. 2019 Jun 28;7:14. doi: 10.1186/s40345-019-0149-9 (PMC6597668; doi:10.1186/s40345-019-0149-9)
Supplement: Supplementary file 1 — Additional file 1. Additional tables. [file 40345_2019_149_MOESM1_ESM.docx]

**Neuropsychological profiles of adult bipolar disorder patients with and without comorbid attention-deficit hyperactivity disorder**

Additional Tables 1a, 1b, & 2.

sTable 1a. Clinical characteristics of the bipolar disorder group without childhood ADHD (BD-cADHD), the bipolar disorder group with childhood ADHD (BD+cADHD), and healthy controls (HC)

|  | BD-cADHD  (*n* = 58-66*) | BD+cADHD  (*n* = 24-32*) | HC  (*n* = 106-112*) | *P*-value | Comparison between groups |
| --- | --- | --- | --- | --- | --- |
| Male, N (%) | 28 (42.4) | 15 (46.9) | 51 (45.5) | .89 |  |
| Female, N (%) | 38 (57.6) | 17 (53.1) | 61 (54.5) |  |  |
|  |  |  |  |  |  |
| Age, mean (SD) | 37.7 (13.6) | 35.2 (11.5) | 37.9 (13.3) | .58 |  |
| Age at first psychiatric symptom, mean (SD) | 20.4 (8.4) | 14.8 (10) |  | .01 |  |
| Age at first affective episode, mean (SD) | 20.9 (8.1) | 16.0 (7.1) |  | .01 |  |
|  |  |  |  |  |  |
| Bipolar I, N (%) | 44 (67.7) | 15 (55.6) |  | .27 |  |
| Bipolar II, N (%) | 21 (32.3) | 12 (44.4) |  |  |  |
| Any comorbid psychiatric diagnosis, N (%) | 36 (58.1) | 20 (76.9) |  | .09 |  |
|  |  |  |  |  |  |
| AUDIT, mean (SD) | 5.5 (4.5) | 6.6 (7) | 6 (3.3) | .54 |  |
| DUDIT, mean (SD) | 0.9 (2.5) | 2.6 (6.6) | 0.1 (0.5) | .00 | BD+cADHD>BD-cADHD, BD+cADHD> HC |
|  |  |  |  |  |  |
| MADRS, mean (SD) | 4.6 (4) | 3.9 (3.3) |  | .40 |  |
| YMRS, mean (SD) | 1.3 (2.1) | 2 (2.7) |  | .16 |  |
|  |  |  |  |  |  |
| **No of mood episodes, mean (SD)** |  |  |  |  |  |
| Mania | 1.9 (2.7) | 1.7 (2.9) |  | .81 |  |
| Hypomania | 4.3 (7.7) | 7.3 (11.8) |  | .16 |  |
| Mixed | 0.4 (1.8) | 6 (10.4) |  | .01 |  |
| Depressive | 8.9 (10.9) | 16 (21.1) |  | .11 |  |
|  |  |  |  |  |  |
| History of attempted suicide or self-harm, N (%) | 22 (34.4) | 13 (48.1) |  | .22 |  |
|  |  |  |  |  |  |
| **Pharmacological treatment, N (%)** |  |  |  |  |  |
| Lithium | 46 (70.8) | 11 (36.7) |  | .00 |  |
| Other mood stabilizers | 13 (20) | 15 (50) |  | .00 |  |
| Antidepressant | 24 (36.9) | 10 (33.3) |  | .73 |  |
| Antipsychotics | 13 (20) | 5 (16.7) |  | .70 |  |
| Central stimulants | 0 | 0 |  |  |  |
| WAIS-III estimated IQ | 107.9 (16) | 106.4 (10.9) | 113.4 (11.3) | .00 | HC>BD-cADHD, HC>BD+cADHD |
| At least 2 years university education, N (%) | 38 (58.5) | 15 (55.6) | 69 (61.6) | .82 |  |
| Working, N (%) | 47 (73.4) | 18 (66.7) | 102 (91.1) | .00 | HC>BD-cADHD, HC>BD+cADHD |
| Sick-leave days previous 12 months, mean (SD) | 116.7 (145.6) | 91.3 (134.8) | 4.7 (11.7) | .00 | HC<BD-cADHD, HC<BD+cADHD |

* Data were missing for some patients, therefore the N varies.

sTable 1b. Specific psychiatric comorbidities of the bipolar disorder group without childhood ADHD (BD-cADHD) and the bipolar disorder group with childhood ADHD (BD+cADHD).

|  | BD-cADHD  (*n* = 63-65*)  N (%) | BD+cADHD  (*n* = 26-27*)  N (%) | *P*-value |
| --- | --- | --- | --- |
| Premenstrual dysphoric disorder | 13 (20.6) | 6 (22.2) | 0.87 |
| Panic disorder | 18 (27.7) | 10 (37) | 0.37 |
| Agoraphobia | 1 (1.5) | 3 (11.1) | 0.04 |
| Social anxiety disorder | 7 (10.8) | 5 (18.5) | 0.31 |
| Obsessive-compulsive disorder | 5 (7.7) | 4 (14.8) | 0.29 |
| Generalized anxiety disorder | 6 (9.2) | 4 (15.4) | 0.40 |
| Posttraumatic stress disorder | 2 (3.1) | 1 (3.7) | 0.88 |
| Alcohol abuse | 13 (20) | 8 (30.8) | 0.27 |
| Alcohol dependence | 4 (6.2) | 4 (14.8) | 0.18 |
| Substance abuse other than alcohol abuse | 11 (16.9) | 4 (14.8) | 0.80 |
| Anorexia nervosa | 4 (6.2) | 5 (18.5) | 0.07 |
| Bulimia nervosa | 4 (6.2) | 3 (11.1) | 0.41 |
| Any personality disorder | 1 (1.6) | 3 (11.1) | 0.04 |

* Data were missing for some patients, therefore the N varies.

sTable 2. Comparison of neuropsychological test performance of the bipolar disorder group without childhood ADHD (BD-cADHD), the bipolar disorder group with childhood ADHD (BD+cADHD), and healthy controls (HC).

|  | BD-cADHD  (*n* = 56-64*) | BD+cADHD  (*n* = 23-31*) | HC  (*n* = 73-112*) | *F*-value | *P*-value | Comparison between groups |
| --- | --- | --- | --- | --- | --- | --- |
|  | Mean (SD) | Mean (SD) | Mean (SD) |  |  |  |
| WAIS-III: Verbal Comprehension Index | 109.8 (12.9) | 112.5 (10.6) | 112.2 (13) | .84 | .43 |  |
| WAIS-III: Perceptual Organization Index | 109.4 (16.5) | 106.1 (14.4) | 116.1 (15.7) | 6.31 | .00 | HC vs. BD-cADHD  *p* =.03  HC vs BD+cADHD  *p* =.01 |
| WAIS-III: Working Memory Index | 102.2 (14.9) | 95.4 (11) | 108.1 (15.1) | 9.59 | .00 | HC vs BD-cADHD  *p* =.03  HC vs BD+cADHD  *p* <.001 |
| WAIS-III: Processing Speed Index | 99.1 (15.8) | 95.9 (10.9) | 107.8 (12.9) | 13.5 | .00 | HC vs BD-cADHD *p* <.001  HC vs BD+cADHD  *p* <.001 |
| CPT2 Omission errors** | 53.5 (15.8) | 53.9 (16.7) | 49.6 (11.8) | 1.72 | .18 |  |
| CPT2 Comission errors** | 54.2 (9.9) | 54.4 (10.1) | 52.2 (10.9) | .88 | .42 |  |
| ColorWord 4 (Inh/Swi) | 9.9 (3) | 9.5 (3.1) | 11.1 (2.5) | 6.11 | .00 | HC vs BD-cADHD *p* =.02  HC vs BD+cADHD  *p* =.01 |
| Tower Test Total | 11.4 (3.3) | 10.4 (3.7) | 11.9 (2.5) | 2.06 | .13 |  |
| RCFT: Immediate recall | 43.5 (14.2) | 41.6 (16) | 48 (13.4) | 3.55 | .03*** |  |
| Claeson Dahl verbal learning | 46 (12.9) | 48.8 (9.3) | 49 (9.9) | 1.58 | .21 |  |

* Data were missing for some patients, therefore the N varies.
** Higher score indicates better performance on all tests except CPT, where lower score indicates better performance.
***The pairwise comparisons showed no significant differences.
